# Medical Society of Tennessee

**Published:** 1840-11

**Authors:** 


### MEDICAL SOCIETY OF TENNESSEE.

This Society at its meeting in May offered a premium of fifty dollars for the best Essay on ***Bilious Fever***, to be submitted under the following circumstances:

"Dissertations on this subject must be transmitted, post paid, to Samuel Hogg, M. D., Nashville, Tennessee, on or before the 1st Monday in March, 1841.

"Each Dissertation must be accompanied with a sealed packet, on which shall be written some device or sentence, and within shall be endorsed the author's name and place of residence. The same device or sentence is to be written on the dissertation to which the packet is attached.

"All unsuccessful dissertations are deposited with the Corresponding Secretary of the Society, from whom they may be obtained if called for within one year after they have been received."

The Committee appointed to read the Dissertations and award the prize, consists of Drs. Felix Robertson, Thomas R. Jennings, and J. H. Atkinson, of Nashville. Y.

